# Persistent Pathogens Following Minimally Invasive Surgery: Is It Time to Rethink Aldehyde-Based Disinfection?

**DOI:** 10.7759/cureus.93682

**Published:** 2025-10-01

**Authors:** Kshitija Nalawade, Vishakha Kalikar, Avinash Supe, Roysuneel Patankar

**Affiliations:** 1 Department of Digestive Diseases, Zen Multispeciality Hospital, Mumbai, IND; 2 Department of Surgery, Zen Multispeciality Hospital, Mumbai, IND; 3 Department of Gastrosurgery, Zen Multispeciality Hospital, Mumbai, IND

**Keywords:** atypical mycobacteria, deep mesh infection, minimal invasive gastrointestinal surgery, minimally invasive laparoscopy, port site infection

## Abstract

Atypical mycobacterial (ATM) infections have emerged as a persistent problem in minimally invasive surgery (MIS), presenting as delayed surgical site infections (SSIs) that are difficult to diagnose and treat. We retrospectively reviewed 47 patients referred to our center over an 11-year period with delayed port-site complications following laparoscopic procedures performed elsewhere. Our focus was on evaluating the link between ATM infections and sterilization practices in hospitals, while secondarily considering outcomes and implications for the prevention of the same.

The mean age was 43.6 years, with a predominance of women (65.9%). The most common primary procedures were laparoscopic cholecystectomy (27.7%) and tubal ligation (23.4%). Clinical presentations included port-site sinuses (38.3%), nodules (27.7%), abscesses (25.5%), and mesh-plane collections (8.5%). Microbiological evaluation confirmed infections due to *Mycobacterium abscessus*, *M. fortuitum*, and the *M. avium *complex. A strong association was observed between ATM infections and the use of 2.45% glutaraldehyde without standardized protocols, whereas autoclaving appeared protective. Patients were treated with prolonged antibiotic therapy (clarithromycin, linezolid, and/or levofloxacin for 3-6 months), and some required surgical debridement or mesh explantation. Our findings highlight ATM as an under-recognized cause of post-laparoscopic SSIs and emphasize that aldehyde-based disinfection is inadequate against these pathogens. Autoclaving should be the preferred sterilization method, and there is an urgent need for institutional and national guidelines to standardize sterilization protocols, improve early diagnosis, and optimize management.

## Introduction

Laparoscopic surgery has significantly transformed modern surgical practices, providing numerous advantages such as reduced recovery times, smaller incisions, and decreased post-operative discomfort. However, there are associated with post-operative complications such as surgical site infections (SSIs) [1]. The delayed SSIs are commonly observed to be atypical mycobacteria (ATM), which have emerged as a notable complication [2].

ATM are less commonly encountered in clinical practice but pose significant challenges due to their resistance to standard antibiotics and tendency to cause chronic infections [3]. ATM are difficult to treat and respond poorly to antibiotics, leading to prolonged and recurrent infections.

The primary source of these infections is found to be tap water used for washing surgical instruments [2,4]. ATMs colonize the joints of dismantled laparoscopic instruments, forming a biofilm around them, thriving despite conventional decontamination methods and protocols [5]. Infections due to ATM frequently present as delayed or chronic port-site wounds that develop into discharging sinuses or abscesses [6]. Outbreaks caused by ATM are observed due to a breach in asepsis and substandard sterilization of laparoscopic instruments [7,8].

ATM infections require advanced microbiological techniques for diagnosis and management, long-term antibiotic therapy, and sometimes surgical intervention [3,9,10]. The risk of infection is higher in immunocompromised patients or in settings where strict infection control measures are not followed [11].

In spite of an increase in the incidence of SSIs due to ATM, there is still a paucity of guidelines for managing post-ATM infections [12].

The purpose of this case series is to find a possible association between ATM infection and multiple perioperative factors and to recommend future studies and some interim pragmatic steps to reduce SSIs. We have compiled the data of 47 cases and analysed it while considering multiple variables over a period of 10 years, confirming the etiology of post-laparoscopic surgery wound infections caused by ATM.

## Materials and methods

An institution-based retrospective analysis of prospectively collected data of patient demographic, type of surgery performed, time since surgery, symptomatology and presentation of patients was collected and multivariate analysis of the data was conducted from October 2013 to October 2024, spanning an 11-year period.

Patients who were operated on for minimally invasive surgeries elsewhere and were referred to our centre with symptoms of delayed port-site wound healing, breakdown of initially healed wounds, erythema of surrounding skin, discharge from the wounds, nodules in or around the vicinity of the wounds, and unexplained low-grade fever, sinus with persistent discharge, or localised collection.

Patient data was categorised in the format of an Excel sheet. The categories, like the name of surgery performed, the time duration of surgery, the type of sterilisation procedure used, and the concentration of glutaraldehyde solution (Cidex™, Advanced Sterilization Products, Irvine, USA), if used, were mentioned. In case of autoclave use, the time for which the instruments were autoclaved was also mentioned. All wounds were categorised in port-site sinuses, nodules, erythema, or collection in the mesh plane if mesh placement was done. The wounds were examined for signs of underlying pockets of collections. In discharging sinuses, culture swabs were taken for sensitivity testing. Imaging studies such as ultrasound, CT scan, or MRI were performed to assess potential collections in the abdominal wall, wherever suspected.

Wound discharge and scraping from sinuses were subjected to smear studies using Gram stain, Leishman stain, and Ziehl-Neelsen (ZN) stain. Upon identification of acid-fast bacilli (AFB), Polymerase Chain Reaction (PCR) specific for *Mycobacterium *species* *and subspecies, along with culture tests for tubercular species identification, were conducted.Tissues obtained during debridement of any infected site or excised tissues and prosthetics were subjected to histopathological examination and culture tests for mycobacteria, along with PCR tests.

Patients with confirmed infections were initiated on second-line anti-tubercular therapy, consisting of clarithromycin (500 mg twice daily) and linezolid (600 mg twice daily), with or without levofloxacin (500 mg twice daily). These agents were administered either individually or in combination for the duration of three to six months. In case of non-responders, debridement of the infected tissue, drainage of the abscess, and explantation of the mesh were done.

**Figure 1:**
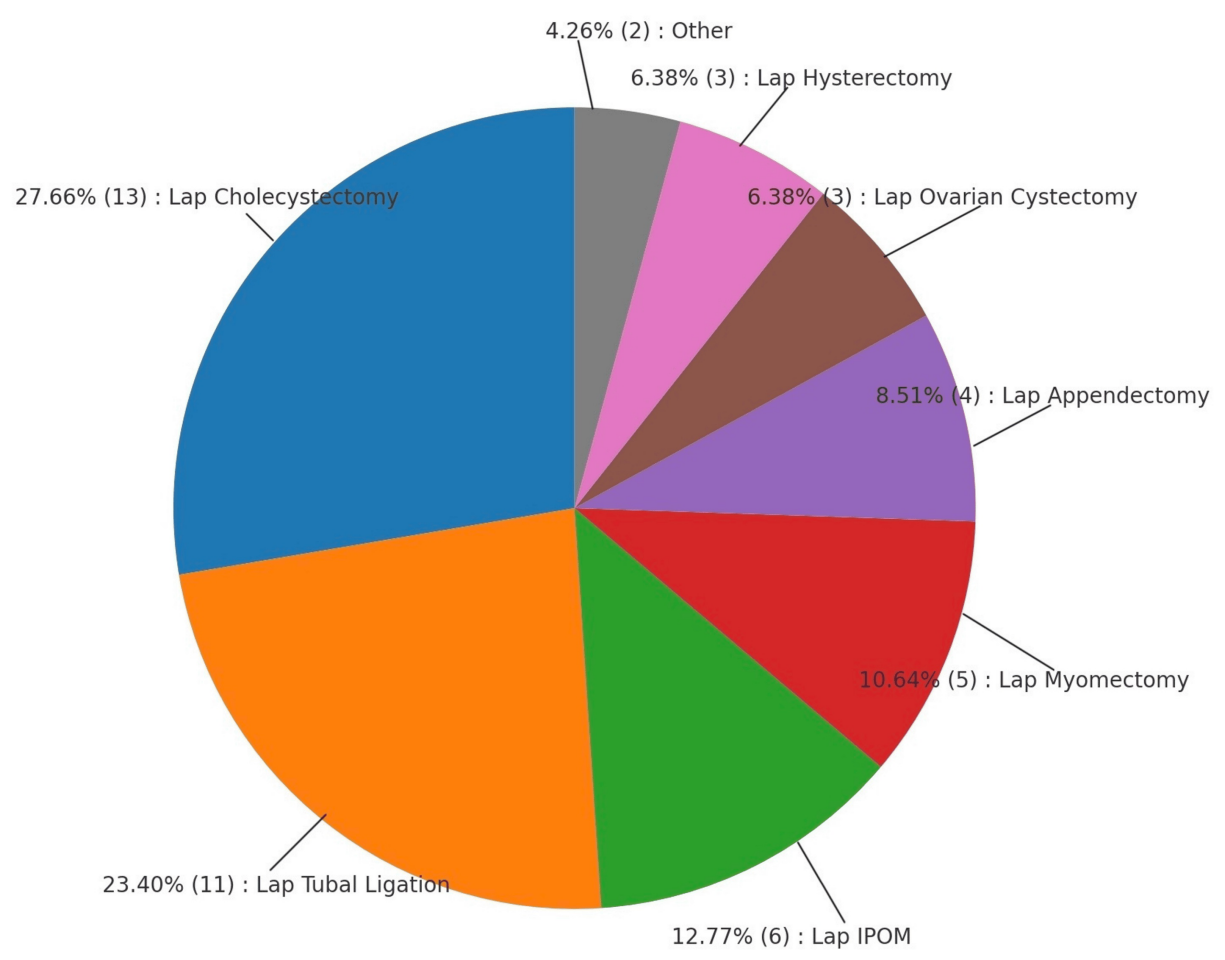
Distribution of laparoscopic procedures IPOM: intraperitoneal onlay mesh

**Figure 2:**
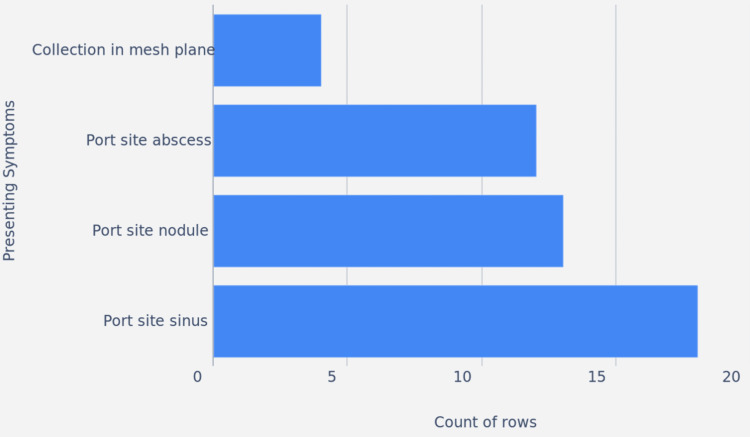
Distribution of symptoms and number of patients affected by it

**Figure 3:**
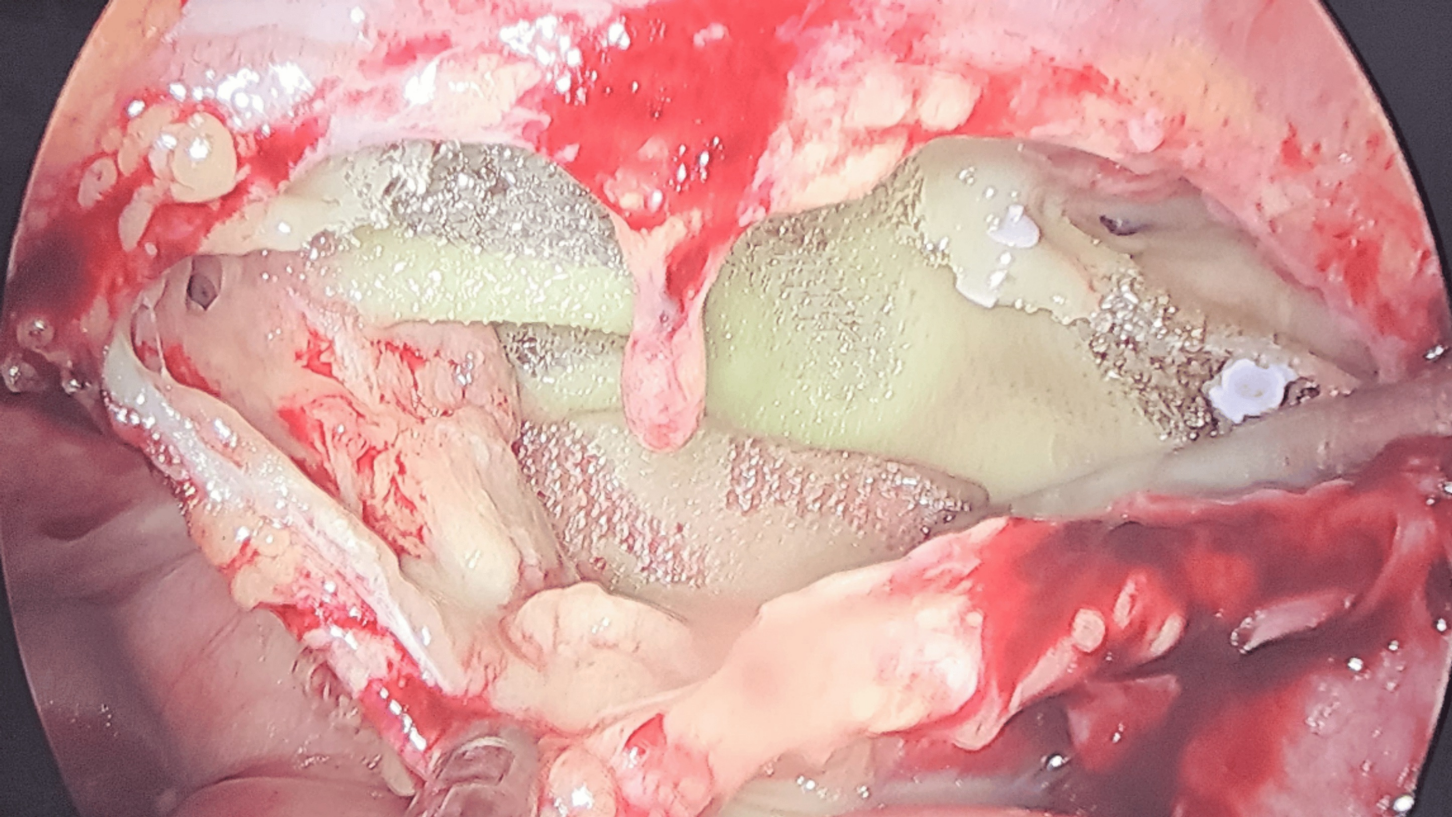
Intraoperative appearance of an infected transabdominal preperitoneal (TAPP) mesh

Figure 1 shows the distribution of surgical procedures, and Figure 2 and Table 1 show the distribution of symptoms and the number of patients affected by them. Figure 3 shows the intraoperative picture of an Infected mesh in a patient operated for trans-abdominal preperitoneal meshplasty.

**Table 1:** Distribution of symptoms

Symptoms	Number of patients affected
Collection in mesh plane	4
Port site abscess	12
Port site nodule	13
Port site sinus	18

## Results

Demographic characteristics and surgical profile of patients

A total of 47 patients who developed port-site complications following laparoscopic surgeries performed at peripheral centers were included in this study. The mean age of the patients was 43.6 years, with a standard deviation of ±11.5 years, indicating a predominantly middle-aged cohort. A female predominance was observed, with 31 patients (65.9%) being women and 16 patients (34.1%) being men. This gender distribution is reflective of the types of laparoscopic procedures performed, many of which were gynecological in nature.

Distribution of laparoscopic procedures

The most common procedure leading to referral for delayed wound complications was laparoscopic cholecystectomy (13 patients; 27.7%), followed by laparoscopic tubal ligation (11 patients; 23.4%) and intraperitoneal onlay mesh (IPOM) repair (6 patients; 12.8%). Other procedures included laparoscopic myomectomy (5 cases; 10.6%), appendectomy (4 cases; 8.5%), ovarian cystectomy (3 cases; 6.4%), hysterectomy (3 cases; 6.4%), and transabdominal preperitoneal (TAPP) hernioplasty (2 cases; 4.3%).

Clinical presentation of port-site infections

Patients presented with variable symptoms, most frequently port-site sinus formation (18 patients; 38.3%), followed by port-site nodules (13 patients; 27.7%), abscesses (12 patients; 25.5%), and collections in the mesh plane (4 patients; 8.5%). Many patients also reported delayed wound healing, persistent low-grade fever, or complete breakdown of initially healed surgical incisions.

Sterilisation methods and risk stratification

A significant portion of the cases were associated with suboptimal sterilisation protocols, particularly in centres where standard autoclaving of laparoscopic instruments was not practiced. Patients from the non-autoclave group demonstrated a markedly higher incidence of port-site infections due to atypical mycobacteria (ATM). Statistical analysis revealed that the odds of developing ATM infection were significantly higher in the non-autoclave group compared to the autoclave group, suggesting a strong association between inadequate instrument sterilisation and infection risk.

Furthermore, the study identified a noteworthy correlation between the use of glutaraldehyde (Cidex)as a sterilising agent and the occurrence of ATM infections. Instruments soaked in glutaraldehyde, especially without strict adherence to contact time and concentration standards, were found to be ineffective in eliminating mycobacteria. This points to a potential iatrogenic source of infection stemming from lapses in sterilisation practices at peripheral centres.

Table 2 provides a comprehensive overview of symptom distribution stratified by sterilisation method, offering insight into the comparative risk profiles of different sterilisation protocols.

**Table 2:** Distribution of symptoms by sterilisation method

Technique	Collection in mesh plane	Port-site sinus	Port-site abscess	Port-site nodule
Autoclave	1	2	1	1
Non-autoclave	3	16	11	12
Odds ratio	0.33	0.12	0.09	0.08

## Discussion

The recent rise in minimal access surgery has highlighted previously unrecognised infections caused by ATM due to the high incidence of surgical infections that do not respond to conventional antibiotics [8]. ATM infections present diagnostic challenges due to their varied presentations and a general lack of awareness. Numerous studies indicate that suboptimal sterility during operations, the use of tap water to clean instruments before autoclaving, and the use of 2.45% gluteraldehyde solution are major contributing factors [7,8,13,14].

The most frequently identified species are *M. abscessus*, *M. fortuitum*, and the *M. avium *complex. Clinical manifestations vary, with most patients presenting with erythema, swelling, and tenderness at the port site. Severe cases may involve abscesses, chronic draining sinuses, or even systemic involvement. Symptoms can emerge from weeks to months following surgery, with some infections being subacute or chronic. Upon encountering an ATM infection, a tissue sample should be sent for Mycobacterium Growth Indicator Tube (MGIT) culture** **and tuberculosis PCR, as these are the gold standards in culture and can identify the specific ATM species causing the infection. In spite of these, the diagnosis of ATM remains challenging. Acid-fast bacilli (AFB) staining and mycobacterial cultures are commonly used diagnostic tools, though they offer varying sensitivity and specificity. Polymerase chain reaction (PCR) and DNA sequencing are more effective, particularly when cultures fail to provide definitive results. In certain outbreaks, molecular epidemiological techniques track infection sources.

Our retrospective data compilation showed that the majority of patients had atypical mycobacterial infections. In telephonic interviews, the referring surgeons confirmed the use of 2.45% glutaraldehyde solution for immersion of instruments before reusing them. The time duration for immersion was not standardised. In spite of the limited data size, we were able to conclude that substandard sterilization of laparoscopic surgical tools and improper use of the disinfectant glutaraldehyde solution (Cidex solution) were shown to result in a higher incidence of ATM contamination and infection.

Management of atypical mycobacterial port-site infections involves a combination of antibiotic therapy and, in some cases, surgical intervention. First-line antibiotics typically include oral linezolid, clarithromycin, and ciprofloxacin [15,16]. Severe cases may necessitate surgical debridement or drainage of abscesses [17,18,19]. Due to the chronic and recurrent nature of these infections, long-term therapy is common.

Preventive strategies focus on stringent infection control measures. Recommended protocols include strict sterilisation practices like autoclaving and disinfection of instruments. Regular surveillance and monitoring of hospital environments. 

We recommend that hospitals implement routine surveillance for ATM infections, particularly in patients undergoing laparoscopic surgery. Strict sterilisation procedures and proper handling of surgical instruments are essential to prevent contamination. They must avoid using gluteraldehyde for disinfection of laparoscopic instruments and telescopes. If used, then to be used with aldehyde indicator pads to analyze the efficiency glutaraldehyde disinfectant solution. In case of spread of infection detected then timely recognition and early intervention are key to effectively managing ATM infections.

We would like to clarify that, like many retrospective studies, ours is subject to certain limitations. This being a single-center study, its findings may not be fully generalizable. The possibility of referral bias exists as our cohort primarily included complicated or persistent cases. The relatively small sample size further restricts the strength of statistical comparisons and may mask subtle trends. Additionally, follow-up was incomplete for some patients, which may have influenced the assessment of long-term outcomes. Despite these constraints, the study draws attention to an important infection-control concern that warrants broader investigation through multicenter prospective studies with larger sample sizes, standardized diagnostic approaches, and uniform follow-up protocols.

## Conclusions

Atypical mycobacterial infections, though uncommon, are an increasingly recognized cause of surgical site complications following minimally invasive surgery. These infections present diagnostic and therapeutic challenges due to their delayed onset, resistance to standard antibiotics, and frequent need for combined medical and surgical management. Our findings demonstrate a strong association between ATM infections and inadequate sterilization practices, particularly the use of glutaraldehyde without standardized protocols. Strict adherence to autoclaving for laparoscopic instruments, routine surveillance, and early diagnostic interventions are essential to reduce this burden. There is an urgent need to develop national and institutional guidelines to standardize sterilization protocols and streamline the management of ATM-related surgical site infections.

There is a need to develop guidelines to standardise, simplify, and direct the management of SSI and mesh infections caused by ATM.
